# Granulin in Frontotemporal Lobar Degeneration: Molecular Mechanisms of the Disease

**DOI:** 10.3389/fnins.2019.00395

**Published:** 2019-04-25

**Authors:** Zemfira N. Karamysheva, Elena B. Tikhonova, Andrey L. Karamyshev

**Affiliations:** ^1^Department of Biological Sciences, Texas Tech University, Lubbock, TX, United States; ^2^Department of Cell Biology and Biochemistry, Texas Tech University Health Sciences Center, Lubbock, TX, United States

**Keywords:** frontotemporal lobar degeneration (FTLD), granulin, disease-causing mutations, protein quality control, protein targeting and transport, signal peptide, signal recognition particle (SRP), RNA degradation

Frontotemporal lobar degeneration (FTLD) is a pathological process characterized by severe atrophy in the frontal and temporal lobes of the brain (Mackenzie et al., 2011). There are three major clinical syndromes in FTLD: behavioral variant of frontotemporal dementia (bvFTD), nonfluent variant of primary progressive aphasia (nfvPPA), and semantic variant of PPA (svPPA) (Gorno-Tempini et al., 2011; Rascovsky et al., 2011). bvFTD is the most common among three (Hernandez et al., 2018). It is associated with changes in personality and behavior accompanied with language deficits at later stages. In rare cases, FTLD subtypes may be associated with motor neuron disease worsening the patient survival time (Olney et al., 2005). FTLD also includes the clinical presentations of progressive supranuclear palsy (PSP) and corticobasal degeneration (CBD), that are associated with parkinsonism, and other clinical features. PSP and CBD account for about 20–30% of patients in FTLD (Park and Chung, 2013). Unfortunately, there is no significant progress achieved in development of effective treatments for FTLD and current treatment options are purely symptomatic (Hodges and Piguet, 2018).

The pathological changes found in FTLD are very heterogenous in their nature. FTLD can be divided in three main histological subtypes according to the accumulation of neuronal protein inclusions (Mackenzie et al., 2011). The most common disease is characterized by the presence of inclusions containing the trans-activation response DNA-binding protein-43 (TDP-43) which is found to be abnormally phosphorylated and ubiquitinated in patients (Neumann et al., 2006). This subtype of pathology is classified as FTLD-TDP (Mackenzie et al., 2011). The second pathological subtype, FTLD-tau, includes cases with inclusions consisting of abnormally phosphorylated microtubule associated protein tau (Cairns et al., 2007). The third subtype, FTLD-FET, contains fused in sarcoma (FUS) RNA-binding protein, Ewing's sarcoma protein (EWS), and TATA-binding protein associated factor 15 (TAF15) in the pathological inclusions (Mackenzie and Neumann, 2012). About 40% of FTLD cases are familial and about 10% of cases exhibit autosomal dominant inheritance (Bang et al., 2015). Mutations in *GRN* (Baker et al., 2006; Cruts et al., 2006), *MAPT* (Hutton et al., 1998), *CHMP2B* (Skibinski et al., 2005), *VCP* (Watts et al., 2004), and *C9orf72* (Renton et al., 2011) have been found associated with the disease. The most common known genetic causes of FTLD are connected with mutations in *GRN, MAPT*, and *C9orf72* genes (Cruts et al., 2006; Gass et al., 2006, 2012; Mori et al., 2013; Hodges and Piguet, 2018). In this article we focus on progranulin (PGRN protein encoded by *GRN* gene) role in FTLD. Patients with progranulin mutations have ubiquitin and TDP-43 positive pathological inclusions (Baker et al., 2006; Cruts et al., 2006). In addition to its role in neurodegenerative diseases PGRN is also implicated in epithelial ovarian cancer and its level is highly elevated in various tumors (He and Bateman, 2003). It also has a role in metabolic diseases and its excess is associated with obesity and insulin resistance (Matsubara et al., 2012). PRGN is a multifunctional protein involved in regulation of many cellular processes including angiogenesis, cell proliferation, inflammation, tissue remodeling, and wound repair (Nguyen et al., 2013).

PRGN is encoded by *GRN* gene that is located on chromosome 17q21 and consists of 13 exons with the Kozak sequence present in the second exon (Bhandari et al., 1992; Cruts and Van Broeckhoven, 2008) (Figure 1A). It encodes 593 amino acid long precursor protein with a predicted molecular mass of 63.5 kDa. PRGN contains a signal peptide (also known as a signal sequence) at the N-terminus to mediate its secretion, followed by 7.5 highly conserved cysteine-rich tandem repeats called granulins. Granulins are separated by divergent linker sequences. Cleavage of the signal peptide generates mature protein that is heavily glycosylated and migrates as 88 kDa protein. This protein is further processed by the cleavage at the linker regions to produce 6 kDa granulins or linked combinations of granulins (Cenik et al., 2012; Gass et al., 2012) (Figure 1B). PGRN does not have clear consensus sequence for protease cleavage and is cleaved by multiple intracellular and extracellular proteases such as elastase, proteinase 3, matrix metallopeptidase 12, or by cathepsins in the lysosomes (Gass et al., 2012; Nguyen et al., 2013; Zhou et al., 2017a). Both progranulin and 6 kDa granulins are shown to exist *in viv*o, however, their biological functions in the cell are not very clear. Recent data suggest that progranulin may be involved in anti-inflammatory activities through modulation of the TNF signaling while granulins are proinflammatory (Tang et al., 2011; Hu et al., 2014). The C-terminus of PRGN is necessary to bind sortilin, a receptor protein regulating intracellular protein trafficking in the Golgi (Hu et al., 2010). Lysosomal targeting of PGRN is carried out by two independent and complementary pathways. The first utilizes sortilin protein, protein trafficking receptor, located in Golgi and cell surface (Hu et al., 2010). The second, sortilin-independent pathway, is mediated by prosaposin (PSAP) through its interaction with mannose 6-phosphate receptor (M6PR) and low-density lipoprotein receptor-related protein 1 (LRP1) (Zhou et al., 2015). PSAP is the precursor of saposin protein essential for lysosomal degradation of glycosphingolipids. The role of PRGN and granulins in lysosome function is poorly understood, however, it has been recently revealed that deficiencies in granulins caused by mutations may play a role in lysosome dysfunction (Holler et al., 2017). Complete loss of PGRN due to homozygous *GRN* mutations was reported as a cause for neuronal ceroid lipofuscinosis (NCL) linking rare lysosomal impairment to neurodegeneration in FTLD (Smith et al., 2012; Gotzl et al., 2016). This disease leads to progressive degeneration of brain and loss of vision due to accumulation of ceroid lipofuscin, a lipid-containing pigment, associated with lysosome dysfunction (Kohlschutter and Schulz, 2009). It was shown that a lack of PGRN leads to decreased level of PSAP in neurons causing NCL (Zhou et al., 2017b). These discoveries suggested that PRGN and PSAP facilitate each other's lysosomal trafficking. Furthermore, studies of lysosome storage diseases from different groups suggested that PRGN might acts as a chaperone of lysosomal enzymes (Jian et al., 2016; Beel et al., 2017). Chaperone functions required direct association of PRGN with lysosomal proteins through granulin E domain and also involved recruitment of HSP70.

**Figure 1:**
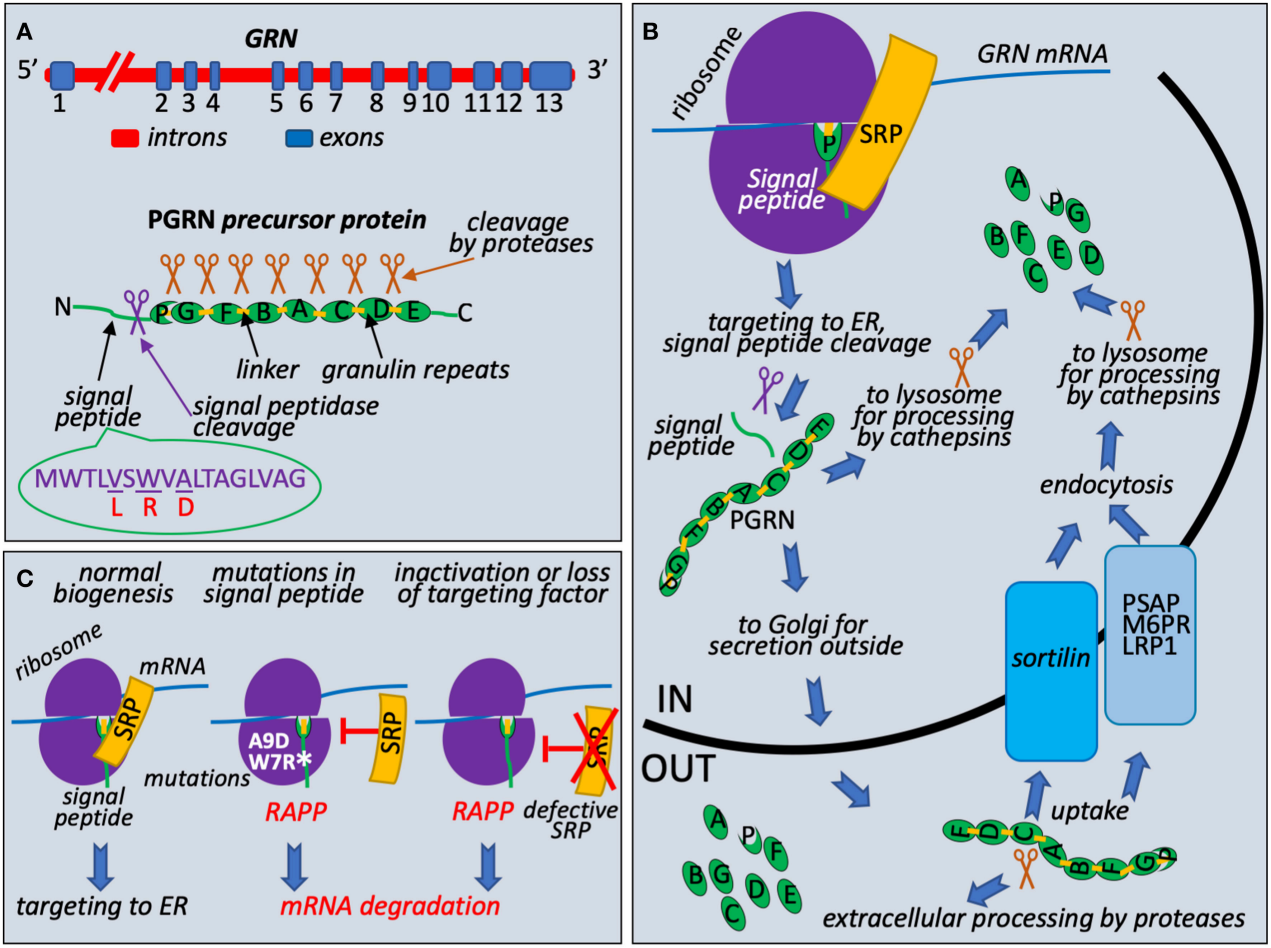
Granulin biogenesis, quality control at the ribosome during its synthesis, and molecular mechanism of FTLD associated with mutations in the signal peptide of the granulin precursor. **(A)** Schematic presentation of *GRN*, precursor protein structure, and known missense mutations in the signal peptide. Progranulin pre-mRNA transcript is synthesized in the nucleus from *GRN* gene in the chromosome 17. It has 13 exons with exons 2–13 containing protein coding region. After splicing, mRNA is exported to cytoplasm for translation. Progranulin precursor (63.5 kDa protein) consists of N-terminal cleavable signal peptide (green line with indicated position of the cleavage by the signal peptidase shown as purple scissors) and 7.5 repeats (green ovals): P (half-repeat, paragranulin), G (granulin 1), F (granulin 2), B (granulin 3), A (granulin 4), C (granulin 5), D (granulin 6), and E (granulin 7). Repeats are connected by linker sequences (light orange boxes). Proteases are shown as brown scissors. Signal peptide sequence presented with known missense mutations L (V5L), R (W7R), D (A9D), corresponding amino acid residues in the wild-type signal peptide are underlined. **(B)** PGRN protein trafficking and processing. During early translation step on the ribosome (purple hemispheres), N-terminal signal peptide of progranulin (green line) is recognized by Signal Recognition Particle (SRP) (shown in orange) and ribosome-nascent chain complex is targeted to ER for signal peptide cleavage (purple scissors), posttranslational modifications, and further processing and transport. Full length protein could be processed to 6 kDa granulins in lysosomes by cathepsins (brown scissors are symbols for all proteases involved in posttranslational processing) or secreted outside and undergo extracellular processing. Uptake of full-length protein is governed by endocytosis with the help of sortilin receptor (blue box) or through alternative PSAP (prosaposin)-dependent pathway with involvement of mannose 6-phosphate receptor (M6PR) and low density lipoprotein receptor-related protein 1 (LRP1) (gray blue box). **(C)** Loss of interaction with targeting factor, SRP, activates RAPP pathway. During normal translational event, PGRN with N-terminal signal sequence is targeted to ER through interaction with SRP. Amino acid sequence of signal peptide and location of reported clinical mutations are shown on **(A)**. When A9D or W7R mutations in signal peptide is detected or SRP is defective or lost, nascent chain is no longer targeted to ER by SRP. It leads to the RAPP pathway activation and degradation of the *GRN* mRNA.

Loss of the PGRN function can occur on the genomic, transcriptional, and posttranscriptional levels (Kleinberger et al., 2013). Mutations in *GRN* are one of the major causes of FTLD and found in 11.2% of patients, therefore progranulin is an important emerging target to develop better treatments (Abella et al., 2017). More than 100 different mutations were identified in the *GRN* gene, and at least 79 pathogenic mutations in 259 families have been associated with FTLD (Cruts et al., 2012) (http://www.molgen.ua.ac.be/FTDmutations/). Most common mutations include nonsense, frameshift and splice site mutations leading to generation of a premature stop codon that activate nonsense-mediated decay (NMD) (Baker et al., 2006; Cruts et al., 2006). Therefore, majority of the mutations are believed to act through a haploinsufficiency mechanism due to mutant mRNA degradation of the one allele and as a result reduced progranulin protein level (Cruts and Van Broeckhoven, 2008). Other mutations include genomic deletions or elimination of the initiation codon for protein synthesis. Loss of the PGRN function can also be mediated by mutations affecting the protein sorting, secretion, proteolytic processing, association with sortilin and cyclin T1, neurite outgrowth, and proinflammatory response (Kleinberger et al., 2013). Some missense and intronic mutations in *GRN* also contribute to the pathogenicity connected to FTLD due to the loss of functional protein (Abella et al., 2017).

Unusual and intriguing molecular mechanism of FTLD that is associated with mutations in progranulin signal sequences was recently discovered (Pinarbasi et al., 2018). Progranulin is a secreted protein and it is synthesized as a precursor with signal peptide (Figure 1A). Signal Recognition Particle (SRP) recognizes signal peptides co-translationally during protein synthesis at the ribosome and targets ribosome nascent complexes to endoplasmic reticulum (ER) membrane for the protein translocation to the ER lumen and further processing and transport outside of the cells (Figure 1B). It is well-established that integrity of the signal peptides is important for protein targeting and transport (Karamyshev et al., 1998; Kalinin et al., 1999; Karamyshev and Johnson, 2005; Nilsson et al., 2015). Despite the absence of the strong amino acid homology between signal peptides of different proteins they have similar organization and contain n-terminal, hydrophobic core or h-region, and c-terminal parts (von Heijne, 1985). Amino acid substitutions that decrease hydrophobicity of the h-region inhibit interaction with SRP (Nilsson et al., 2015). As we recently discovered, the loss of SRP interaction activates the protein quality control pathway named RAPP (regulation of aberrant protein production) leading to mRNA degradation of the defective proteins (Karamyshev et al., 2014; Karamyshev and Karamysheva, 2018). Among more than 100 of different mutations in the progranulin three missense mutations lead to amino acid alterations in the signal peptide hydrophobic core; they are V5L, W7R, and A9D (Gass et al., 2006; Mukherjee et al., 2006; Lopez de Munain et al., 2008; Cruts et al., 2012) (Figure 1A). While V5L and W7R mutations are not well-studied in patients, it was demonstrated that the A9D mutation resulted in decreased *GRN* mRNA and protein levels (Mukherjee et al., 2006, 2008). However, the mechanism of the reduced mRNA level was not clear at that time. Further detailed experimental examination of the PGRN signal peptide mutations showed that W7R and A9D inhibited signal peptide interaction with SRP and pathologically activated the RAPP pathway leading to degradation of the defective *GRN* mRNAs establishing the molecular mechanism of the familial FTLD through mRNA degradation (Pinarbasi et al., 2018) (Figure 1C). Remarkably, the mechanism of *GRN* mRNA degradation was specific to the mutated mRNAs only and did not affect the wild-type *GRN* mRNA when they were co-expressed. The mRNA degradation was initiated by the loss of SRP interaction with the signal peptide due to W7R or A9D mutation. RAPP activation is a unique feature of the pathway—it recognizes defective proteins and degrades their mRNA templates. Interestingly, V5L mutation did not interfere with SRP interactions and did not induce the RAPP pathway, and the mutated mRNA did not degrade, suggesting that the V5L is a benign polymorphism and most likely does not lead to a disease. Analysis of the signal peptide hydrophobicity profiles revealed that W7R or A9D mutations decreased hydrophobicity while V5L did not. This observation may be used for theoretical prediction of the impact of the uncharacterized mutations for RAPP activation and mRNA degradation. Noteworthy, the depletion of SRP54 (one of the six SRP subunits) led to mRNA degradation of the wild-type protein (Figure 1C). This fact suggests that defects in SRP subunits may be a molecular basis of sporadic human diseases. Indeed, it was found recently that several mutations in SRP54 are associated with inherited neutropenia and Shwachman-Diamond-like syndrome (Carapito et al., 2017).

Polypeptide nascent chain interactions at the ribosome are important for proper protein folding, transport, and modification. As it is discussed above, the loss of the SRP signal peptide interaction leads to dramatic consequences: elimination of the defective protein mRNA in the RAPP pathway and as a result to decrease of PGRN protein level and finally to FTLD. Most likely, the induction of the RAPP pathway is not limited to the mutant PGRNs, and may be associated with signal peptide mutations in other secretory proteins leading to the diverse group of the human diseases caused by the pathological RAPP activation.

In conclusion, it seems that the decrease or loss of *GRN* expression in many different familial FTLDs is associated with mRNA degradation, although the nature of the mutations is different. The nonsense, frameshift, and splice site mutations generate premature stop codons that induce NMD, while the mutations in the signal peptide activate RAPP. Regardless of the pathway engaged, the *GRN* mRNA is degraded that may lead to PGRN haploinsufficiency and the disease. These observations open the necessity of deep exploration of the molecular mechanisms of mRNA degradation pathways in neurodegenerative diseases that may eventually lead to development better pharmacological treatments in the future.

## Author Contributions

AK and ZK wrote the manuscript. ET designed and prepared the figure, all authors discussed and edited the manuscript.

### Conflict of Interest Statement

The authors declare that the research was conducted in the absence of any commercial or financial relationships that could be construed as a potential conflict of interest.
